# Study on the Adsorption Characteristics of Methylene Blue by Magnesium-Modified Fly Ash

**DOI:** 10.3390/molecules30050992

**Published:** 2025-02-21

**Authors:** Junxia Zhou, Mengjie Li, Yu Tao, Lanchang Zha

**Affiliations:** 1 School of Civil Engineering, Liaoning Technical University, Fuxin 123000, China; 2 Ordos Institute of Liaoning Technical University, Ordos 017004, China

**Keywords:** magnesium modification, fly ash, adsorption, methylene blue

## Abstract

Aiming at the pollution problem of methylene blue dye wastewater, a new type of methylene blue adsorbent magnesium-modified fly ash (Mg@FA) was prepared by using solid waste fly ash as raw material. The effects of Mg@FA dosage, adsorption time, and methylene blue concentration on the adsorption of methylene blue by Mg@FA and pH values were analyzed. The adsorption characteristics of Mg@FA on methylene blue were investigated by adsorption kinetics, adsorption isotherms, and adsorption thermodynamics, as well as SEM, EDS, XRD, BET, and FTIR. The results showed that when the dosage of Mg@FA was 1.0 g, the adsorption time was 120 min, and the initial concentration of methylene blue was 150 mg/L; the adsorption efficiency of methylene blue by Mg@FA was the highest, which was 95.61%. When the pH of the methylene blue solution was in the range of 7–11, the adsorption efficiency of Mg@FA for methylene blue remained stable at 95.61–98.10%. The adsorption process of methylene blue by Mg@FA follows the second-order kinetic fitting model and Langmuir model. The adsorption of methylene blue by Mg@FA is a spontaneous and endothermic reaction. Mg@FA adsorbs methylene blue through electrostatic interaction and hydrogen bonding. Mg@FA can effectively adsorb methylene blue and promote the waste utilization of fly ash, which provides a promising method for wastewater treatment and fly ash utilization.

## 1. Introduction

With the rapid development of industry, the lack of clean drinking water worldwide is one of the health problems faced by contemporary society [1]. Industrial dye pollution is an important cause of water being undrinkable [2]. At present, dyes are widely used in papermaking, textiles, plastics, leather, and other fields, and the annual output of dyes is about 7 × 10^5^ tons [3]. Among these commonly used dyes, methylene blue (MB) has the characteristics of toxicity, carcinogenicity, strong chemical resistance, and non-biodegradability. It is easy to stimulate the eyes, resulting in difficulty in breathing, vomiting, confusion, and methemoglobinemia [2,3]. MB is often used as a typical dye to evaluate the performance of adsorbents [4]. Huseyin Karaca et al. [5] found that the maximum adsorption capacity of fly ash to methylene blue at 298 K and 398 K was 0.12 mg/g and 0.07 mg/g, respectively. When Anna Witek-Krowiak et al. [6] used fly ash as an adsorbent to remove cationic dyes, it was found that the adsorption isotherm data conformed to the Langmuir model. Fly ash can be used as a cheap adsorbent to remove dyes such as methylene blue, but the adsorption effect is limited [7].

Currently, a large number of studies have focused on modifying fly ash to improve its adsorption performance. Kaman Singh et al. [8] prepared modified fly ash (MFA) using NaOH solution and TiO_2_ and found that MFA exhibited a higher adsorption efficiency for methylene blue dye compared to fly ash. Johaina Alahmad et al. [9] prepared an adsorbent, FAG-TiO_2_, by modifying fly ash with nano-TiO_2_. FAG-TiO_2_ effectively adsorbed methylene blue dye, and the adsorption process followed the Langmuir isotherm model. Chan Juan Li et al. [10] modified fly ash by acid treatment, which increased its maximum removal rate for methylene blue to 97.8%. Li Juan et al. [11] found that the adsorption performance of fly ash was significantly improved after modification with a citric acid solution, with the methylene blue fixation rate reaching 89.39%. Gajera Rutuben et al. [12] prepared TiO_2_-loaded acid-modified fly ash–chitosan composites, and compared to unmodified fly ash composites; this composite material improved the removal rate of methylene blue dye by 10%. Bai Yujie et al. [13] found that acid-modified fly ash (M-FA) had better adsorption capacity for methylene blue than water-washed fly ash (FA). The equilibrium adsorption capacities of FA and M-FA for methylene blue were 4.22 mg/g and 5.98 mg/g, respectively. The above studies indicate that modification methods such as TiO_2_ modification and acid treatment can enhance the ability of fly ash to adsorb methylene blue. This approach promotes environmental sustainability by reducing waste generation and the efficient use of resources [9]. It was also reported that Mg modification can effectively improve the adsorption capacity of the adsorbent on dyes. The maximum adsorption capacities of magnesium oxide-modified diatomite prepared by Ningning Liu et al. [14] for methylene blue and acid orange were 25.02 and 35.13 mg/g, respectively. Yanxin Wei et al. [15] prepared polysilicate magnesium sulfate using MgSO_4_ and polysilicic acid, and its removal rate of Congo red can reach more than 80%. The removal rate of Congo red by modified magnesium ferrite nanoparticles prepared by Supawitch Hoijang et al. [16] was more than 90%. Guo Zhengzheng [17] found that when preparing magnesium-containing zeolite-type calcined diatomite with MgCl_2_ and NaOH as modifiers, the Mg^2+^ in the modifier could replace Al^3+^, thereby improving its adsorption performance for methylene blue. After magnesium modification, its adsorption capacity for methylene blue increased by 67 times. Zhu Guanyu [18] used fly ash as the raw material to prepare magnesium-modified fly ash-based tobermorite and found that Mg could replace some of the Ca in fly ash, forming MgO and Mg(OH)_2_, which enhanced its adsorption capacity for nitrogen and phosphorus. Fly ash is a solid waste residue formed by the high-temperature combustion of coal, mainly composed of SiO_2_, Al_2_O_3_, CaO, Fe_2_O_3_, K_2_O, Na_2_O, and other components [19]. The magnesium modification process can replace some of the metals in fly ash, forming MgO and Mg(OH)_2_, thereby improving its adsorption capacity. The above studies show that Mg modification can effectively promote the adsorption of dyes such as methylene blue, acid orange, and Congo red. However, there are few reports on the adsorption of dyes by Mg modification fly ash at this stage.

Therefore, a new type of methylene blue dye adsorbent magnesium-modified fly ash (Mg@FA) was prepared by using fly ash and MgCl_2_ as raw materials. Based on batch experiments, the effects of magnesium-modified fly ash dosage, adsorption time, methylene blue concentration, and pH values on the adsorption of methylene blue by magnesium-modified fly ash were investigated. Based on the principle of adsorption kinetics, adsorption isotherm, and adsorption thermodynamics, combined with analyses such as SEM, FTIR, and XRD, the mechanism of methylene blue adsorption of Mg modification of fly ash was revealed.

## 2. Results and Analysis

### 2.1. Effects of Mg@FA Dosage, Adsorption Time, Methylene Blue Concentration, and pH Values on Adsorption of Methylene Blue by Mg@FA

**Figure 1:**
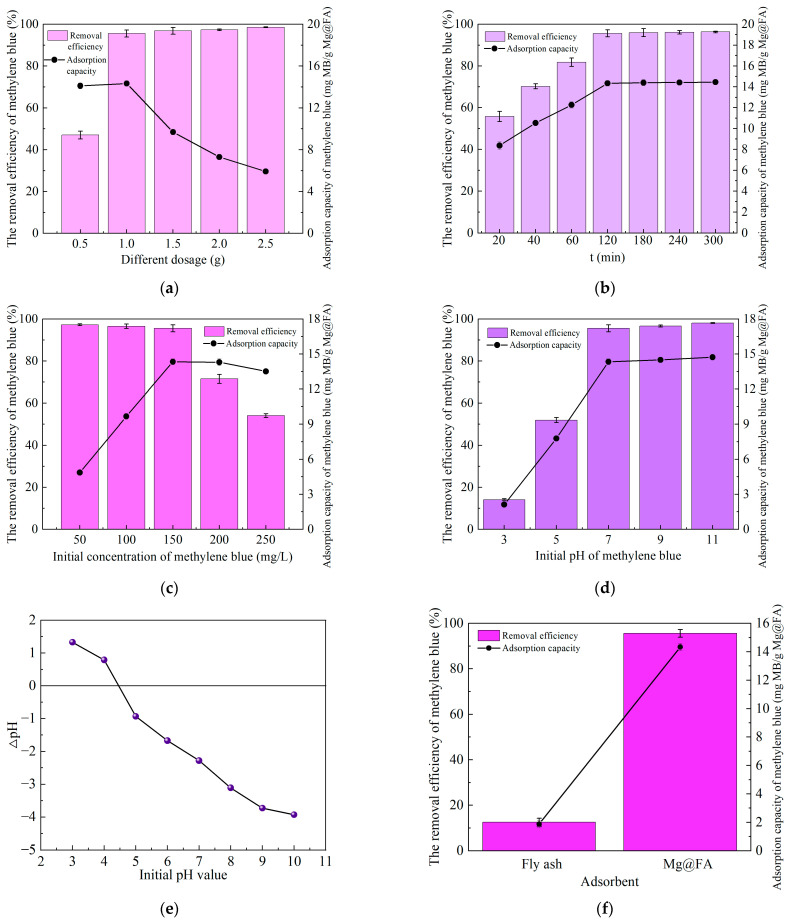
The effect of Mg@FA adsorption of methylene blue. (**a**) The effect of Mg@FA dosage. (**b**) The effect of adsorption time. (**c**) The effect of methylene blue concentration. (**d**) The effect of solution pH. (**e**) The point of zero charge (pHpzc) of Mg@FA. (**f**) A comparison of the adsorption effects of fly ash and Mg@FA on methylene blue.

It can be seen from Figure 1a that when the dosage of Mg@FA was 0.5 g, the removal efficiency of methylene blue by Mg@FA was only 47.04%. When the dosage of Mg@FA was 1.0–2.5 g, the removal efficiency of methylene blue by Mg@FA remained stable at 95.61–98.62%. This shows that when 0.5–1.0 g Mg@FA is added, the dosage of Mg@FA is the main factor affecting the adsorption of methylene blue by Mg@FA. With the increase in Mg@FA dosage, the adsorption active sites increase, which is beneficial to the adsorption and removal of methylene blue. When 1.0–2.5 g Mg@FA was added, the dosage of Mg@FA almost did not affect the removal efficiency of methylene blue by Mg@FA. Interestingly, when the dosage of Mg@FA is 0.5–1.0 g, the adsorption capacity of Mg@FA for methylene blue is as high as 14.11–14.34 mg/g. However, when the dosage of Mg@FA was 1.0–2.5 g, the adsorption capacity of Mg@FA for methylene blue gradually decreased from 14.34 mg/g to 5.92 mg/g with the increase in Mg@FA dosage. This is mainly due to the limited number of methylene blue dyes in the solution. When the dosage of Mg@FA is large, there are too many adsorption active sites, and almost all methylene blue is removed. When the dosage of Mg@FA is 1.0 g, the adsorption effect of Mg@FA on methylene blue is better, and the dosage of Mg@FA is lower.

It can be seen from Figure 1b that with the increase in reaction time, the adsorption removal efficiency and adsorption capacity of Mg@FA for methylene blue increased first and then stabilized. When the adsorption time was 20 min, the adsorption removal efficiency and adsorption capacity of Mg@FA for methylene blue were only 55.79% and 8.37 mg/g, respectively. When the adsorption time was 120 min, the adsorption removal efficiency and adsorption capacity of Mg@FA for methylene blue were relatively high, at 95.61% and 14.34 mg/g, respectively. When the adsorption time was 120–300 min, the adsorption of methylene blue by Mg@FA tended to be stable. When Mg@FA adsorbs methylene blue for 120 min, the adsorption and fixation effect of methylene blue is better.

It can be seen from Figure 1c that when the initial concentration of methylene blue is 50–150 mg/L, the removal efficiency of methylene blue by Mg@FA remains stable at 95.61–97.28%. When the initial concentration of methylene blue was 150–250 mg/L, the removal efficiency of methylene blue by Mg@FA decreased from 95.61% to 54.04%. However, the adsorption capacity of Mg@FA for methylene blue increased first and then stabilized with the increase in methylene blue concentration. When the initial concentration of methylene blue was 50–150 mg/L, the adsorption capacity of Mg@FA to methylene blue increased from 4.86 mg/g to 14.34 mg/g. When the initial concentration of methylene blue was 150–250 mg/L, the adsorption capacity of Mg@FA to methylene blue remained stable at 13.51–14.34 mg/g. This is because when the dosage of adsorbent Mg@FA is consistent, the number of adsorption active sites is constant. With the increase in methylene blue concentration, the adsorption of methylene blue by Mg@FA gradually tends to be saturated.

As shown in Figure 1d, when the pH of the methylene blue solution is between 3 and 11, the removal efficiency of methylene blue by Mg@FA first increases and then stabilizes. When the pH of the methylene blue solution is between 3 and 7, the removal efficiency of methylene blue by Mg@FA increases from 14.12% to 95.61%. However, when the pH of the methylene blue solution is between 7 and 11, the removal efficiency of methylene blue by Mg@FA stabilizes between 95.61% and 98.10%. Combined with Figure 1e, it can be seen that the point of zero charge (pHpzc) of Mg@FA is 4.46. When the solution pH is 4.46, Mg@FA is electrically neutral. When the solution pH is less than 4.46, Mg@FA is positively charged, and when the solution pH is greater than 4.46, Mg@FA is negatively charged. Methylene blue is a cationic dye, which is more easily adsorbed by adsorbents with a negative charge or when pH > pHpzc [20]. When the pH of the methylene blue solution is 3, Mg@FA is positively charged, which repels methylene blue, leading to weaker adsorption. When the pH of the methylene blue solution is between 5 and 11, Mg@FA is negatively charged, and the electrostatic attraction formed with methylene blue promotes the adsorption of methylene blue by Mg@FA.

As shown in Figure 1f, under the same experimental conditions (adsorbent dosage 1 g, methylene blue solution volume 100 mL, methylene blue concentration 150 mg/L, 25 °C, 150 r/min, adsorption for 120 min), the adsorption removal efficiency and adsorption capacity of the fly ash used in the experiment for methylene blue were only 12.53% and 1.88 mg/g, respectively. However, the adsorption removal efficiency and adsorption capacity of Mg@FA for methylene blue were 95.61% and 14.34 mg/g, respectively. Compared to untreated fly ash, the Mg@FA prepared in this study increased the removal efficiency of methylene blue by 83.08%, and its adsorption capacity was 7.63 times that of fly ash. This indicates that Mg@FA can effectively enhance the adsorption performance of fly ash for methylene blue.

Table 1 summarizes the adsorption of methylene blue by different adsorbents reported in the previous literature. The data shows that, compared to fly ash [5,21], Mg@FA significantly improves the adsorption capacity for methylene blue. Compared to calcium silicate [22], starch phosphate grafted polyvinyl imidazole [23], and magnetic/activated charcoal/β-cyclodextrin/alginate polymer beads [24], the adsorbent Mg@FA also exhibits good methylene blue adsorption capacity, with the adsorption capacity of Mg@FA for methylene blue increasing by 0.90 mg/g, 9.14 mg/g, and 12.26 mg/g, respectively. However, there is a difference when compared to activated carbon for methylene blue adsorption. Compared to walnut shell-activated carbon [25], almond shell-activated carbon [25], corn cob-derived activated carbon [26], fir wood-derived activated carbon [27], and magnetized corn cobs [28], the adsorption capacity of Mg@FA for methylene blue increased by 10.81 mg/g, 13.01 mg/g, 13.50 mg/g, 13.13 mg/g, and 1.11 mg/g, respectively, showing that Mg@FA exhibits good methylene blue adsorption performance. However, when compared to biochar prepared from date palm seeds [29], Mg@FA shows a relatively weaker adsorption ability for methylene blue. The main reason for this difference is that the biochar prepared from date palm seeds has a stronger adsorption capacity. The above comparison indicates that the adsorbent Mg@FA demonstrates a relatively strong methylene blue removal capacity compared to previous reports, validating the effectiveness of this low-cost adsorbent Mg@FA in the treatment of methylene blue wastewater.

### 2.2. Adsorption Kinetics of Methylene Blue on Mg@FA

**Figure 2:**
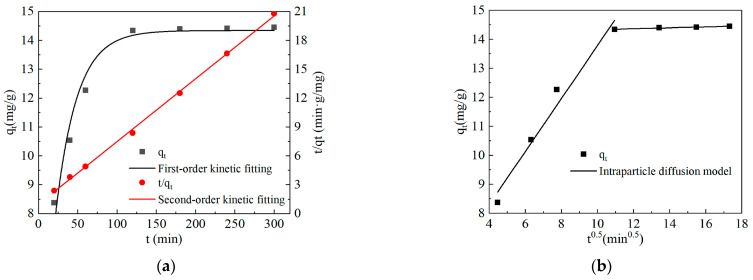
Fitting curve of adsorption kinetics of methylene blue on Mg@FA (**a**) pseudo-first-order kinetic model and pseudo-second-order kinetic model. (**b**) Intraparticle diffusion model.

The adsorption kinetics fitting results of Mg@FA adsorption of methylene blue are shown in Figure 2 and Table 2.

From Figure 2 and Table 2, it can be seen that the second-order kinetic fitting *R*^2^ of Mg@FA adsorption of methylene blue is higher than the first-order kinetic fitting *R*^2^. This indicates that the process of Mg@FA adsorbing methylene blue conforms to the second-order kinetic fitting model. The intraparticle diffusion model was used to fit the adsorption process of methylene blue by Mg@FA in segments. The fitting results show that the adsorption process of methylene blue by Mg@FA can be divided into two stages based on the rate constant *k_p_*, with *k_p_*_1_ > *k_p_*_2_. This indicates that the adsorption process of methylene blue by Mg@FA can be divided into surface diffusion and intraparticle diffusion stages. In the first stage, there are many free adsorption sites on the surface of Mg@FA, allowing methylene blue to be rapidly adsorbed on the surface of Mg@FA. In the second stage, most of the adsorption sites on the surface of Mg@FA are occupied by methylene blue, and only a small amount of methylene blue diffuses into the inner layers of Mg@FA for adsorption. The intraparticle diffusion model fitting lines for the adsorption of methylene blue by Mg@FA do not pass through the origin, indicating that intraparticle diffusion is not the only rate-limiting step during the adsorption of methylene blue by Mg@FA.

### 2.3. Adsorption Isotherms and Adsorption Thermodynamics of Methylene Blue on Mg@FA

**Figure 3:**
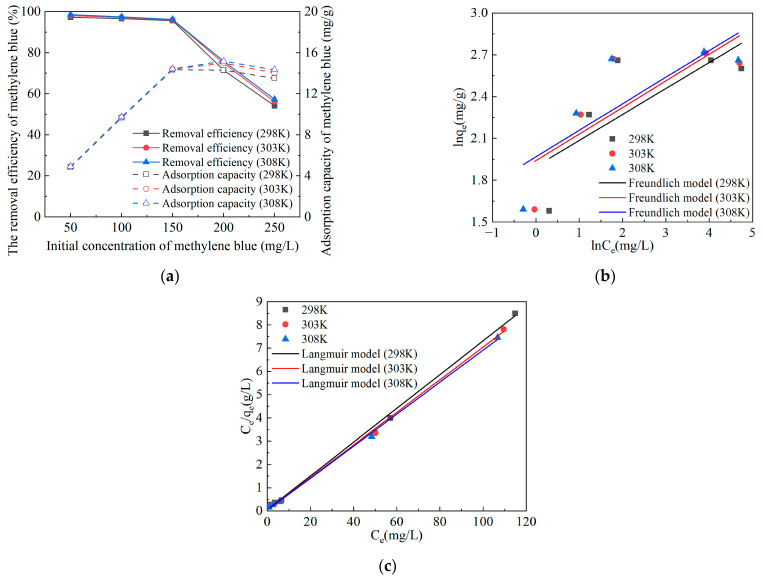
Freundlich and Langmuir fitting curves for adsorption of methylene blue by Mg@FA. (**a**) Adsorption of methylene blue by Mg@FA under different temperature conditions. (**b**) Freundlich fitting curve for adsorption of methylene blue by Mg@FA. (**c**) Langmuir fitting curve for adsorption of methylene blue by Mg@FA.

The adsorption of methylene blue by Mg@FA under different temperature conditions is shown in Figure 3a. The adsorption isotherm fitting results of Mg@FA adsorbing methylene blue are shown in Figure 3b,c and Table 3.

As shown in Figure 3a, when the temperature is 298 K, 303 K, and 308 K, the adsorption of methylene blue by Mg@FA is similar. When the initial concentration of methylene blue is between 50 and 150 mol/L, temperature changes have little effect on the adsorption of methylene blue by Mg@FA. However, when the initial concentration of methylene blue is between 150 and 250 mol/L, as the temperature increases, the removal efficiency and adsorption capacity of Mg@FA for methylene blue also increase. It can be seen from Figure 3b,c and Table 3 that the Langmuir model fitting *R*^2^ of Mg@FA adsorption of methylene blue is greater than the Freundlich model fitting *R*^2^. This shows that the process of Mg@FA adsorbing methylene blue is more in line with the Langmuir model and follows the Langmuir monolayer adsorption law. By calculating the adsorption equilibrium constant *R_L_* (*R_L_* = 1/(1 + *c*_0_ × *K_L_*)), it can be seen that the *R_L_* of Mg@FA adsorbing methylene blue satisfies 0 < *R_L_* < 1, which proves that Mg@FA is beneficial to the adsorption process of methylene blue. To further investigate the thermodynamic properties of the reaction process, Gibbs free energy ∆G (kJ/mol), enthalpy change ∆H (kJ/mol), and entropy change ∆S (kJ/(mol·K)) were determined using the Van’t Hoff isothermal equation and Gibbs formula, with the results shown in Table 4. At the experimental temperatures (298 K, 303 K, 308 K), ∆G is negative, and as the temperature increases, ∆G decreases. The value of ∆H = 36.41429 kJ/mol is positive. This indicates that the adsorption reaction of Mg@FA for methylene blue is spontaneous and endothermic, and increasing the temperature is favorable for the adsorption of methylene blue by Mg@FA. The value of ∆S = 125.08455 kJ/(mol·K) is positive, suggesting that the disorder at the solid–liquid interface increases during the adsorption process.

### 2.4. Mechanism Analysis of Methylene Blue Adsorption by Mg@FA

**Figure 4:**
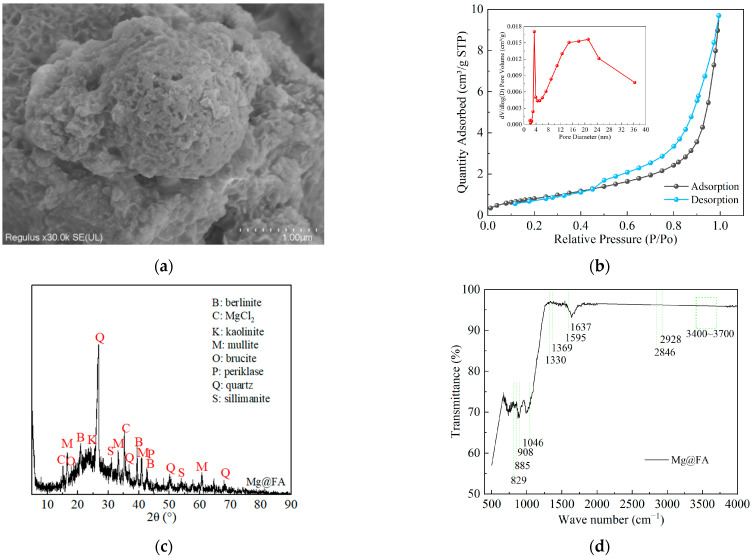

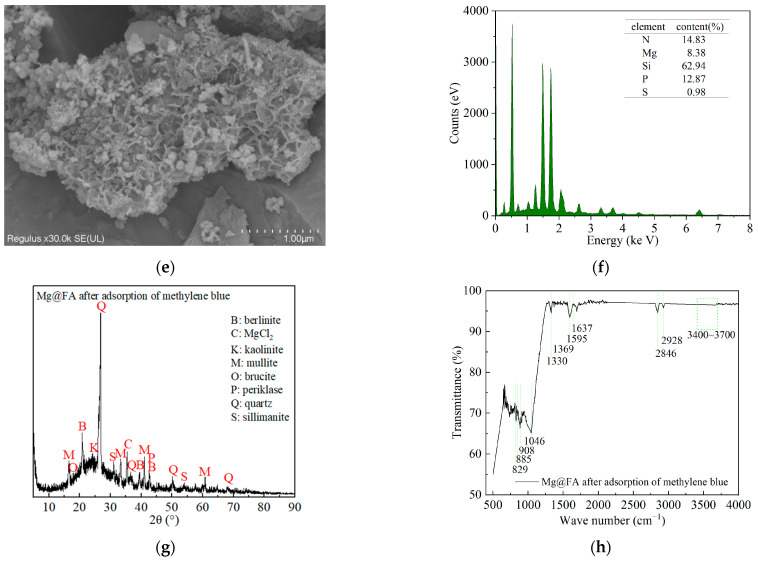
SEM, XRD, BET, EDS, and FTIR of Mg@FA adsorption of methylene blue. (**a**) SEM of Mg@FA. (**b**) N_2_ adsorption–desorption isotherm and corresponding pore size distribution of Mg@FA. (**c**) XRD of Mg@FA. (**d**) FTIR of Mg@FA. (**e**) SEM of Mg@FA after adsorption of methylene blue. (**f**) EDS of Mg@FA after adsorption of methylene blue. (**g**) XRD of Mg@FA after adsorption of methylene blue. (**h**) FTIR of Mg@FA after adsorption of methylene blue.

Mg@FA and Mg@FA after the adsorption of methylene blue were analyzed using SEM, EDS, XRD, BET, and FTIR. SEM (Regulus 8100, Hitachi High-Technologies Group, Tokyo, Japan) and EDS (Bruker Nano GmbH, Berlin, Germany) were used to analyze the morphology and elemental content of the materials. XRD (Smart Lab 9, Rigaku Corporation, Tokyo, Japan) was used to analyze the mineral composition of the materials, with a detection range of 5° to 90°. FTIR (Nicolet iS5, Thermo Fisher Scientific, Waltham, MA, USA) was used to analyze the functional groups of the materials, with a testing range from 4000 cm^−1^ to 500 cm^−1^. The specific surface area and porosity of the materials were analyzed using an automatic surface area and porosity analyzer (ASAP 2460, Micromeritics Instrument Corporation, Atlanta, GA, USA). The results of SEM, EDS, XRD, BET, and FTIR analyses are shown in Figure 4.

The detection results of Mg@FA are shown in Figure 4a–d. As shown in Figure 4a, the surface of Mg@FA has a large number of raised prismatic structures, which are interconnected to form a network-like structure covering the surface of fly ash. Figure 4b shows the N_2_ adsorption–desorption isotherm and corresponding pore size distribution of Mg@FA. Based on the data in Figure 4b, the specific surface area of Mg@FA was calculated to be 3.1563 m^2^/g, and the total pore volume was 0.014745 cm^3^/g using the Brunauer–Emmett–Teller (BET) method. The average pore diameter of Mg@FA was found to be 14.9825 nm using the Barrett–Joyner–Halenda (BJH) theory for mesopore analysis. This indicates that the surface of Mg@FA has pores, with mesopores being the dominant type. As shown in Figure 4c, the main characteristic diffraction peaks in Mg@FA include mullite, quartz, kaolinite, sillimanite, berlinite, MgCl_2_, brucite, and periclase. The characteristic diffraction peaks of MgCl_2_, brucite, and periclase appearing in Mg@FA indicate that Mg was successfully added to the surface of the fly ash. Figure 4d shows that Mg@FA exhibits bending vibration peaks of water molecules at 1637 cm^−1^ and O-H stretching vibration peaks of free hydroxyl groups in the range of 3400–3700 cm^−1^ [30]. This suggests that Mg@FA contains a large amount of OH^−^, which is conducive to promoting the adsorption of methylene blue by Mg@FA.

The detection results of Mg@FA after adsorbing methylene blue are shown in Figure 4e–h. As shown in Figure 4e, after adsorbing methylene blue, the surface of Mg@FA still exhibits the network-like structure formed by the raised prismatic structures. From Figure 4f, it can be seen that after adsorbing methylene blue, the contents of N, Mg, Si, P, and S in Mg@FA are 14.83%, 8.38%, 62.94%, 12.87%, and 0.98%, respectively. Among them, N and S mainly originate from methylene blue, further confirming that Mg@FA has successfully adsorbed methylene blue. As shown in Figure 4g, after adsorbing methylene blue, the intensity of some diffraction peaks increases. It was reported that after adsorbing methylene blue, the peak intensity significantly increases, further indicating that the dye molecules are bound to the active sites of the adsorbent [31]. From Figure 4h, it can be seen that the Mg@FA after adsorption of methylene blue corresponds to the C=N stretching peak and the -CH_3_ symmetric bending vibration peak of methylene blue at 885 cm^−1^ and 1330 cm^−1^, respectively [31]. At 829 cm^−1^ and 908 cm^−1^, respectively, the out-of-plane bending vibration peak of the benzene ring C-H and the stretching vibration peak of the C-C skeleton occur [30]. At 1046 cm^−1^ and 1595 cm^−1^, the tensile vibration peak of C=S of methylene blue and the bending vibration peak of N-H correspond, respectively [32]. The unique vibrations at 2928 cm^−1^ and 2846 cm^−1^ correspond to the -N(CH_3_)_2_^+^ stretching vibration peak of methylene blue [33]. These new absorption peaks indicate that methylene blue is adsorbed by Mg@FA. In particular, the -N(CH_3_)_2_^+^ group in methylene blue is positively charged, and the membrane surface of Mg@FA is negatively charged, which indicates that Mg@FA can adsorb methylene blue through electrostatic interaction [31,33]. In addition, the signal intensity of the hydroxyl vibration peak corresponding to 3400–3700 cm^−1^ of Mg@FA after adsorption of methylene blue was weakened, indicating that hydrogen bonds were formed between Mg@FA and methylene blue [33]. Therefore, Mg@FA adsorbs methylene blue through electrostatic interaction and hydrogen bonding.

### 2.5. Mechanism Analysis of Methylene Blue Adsorption by Mg@FA

**Figure 5:**
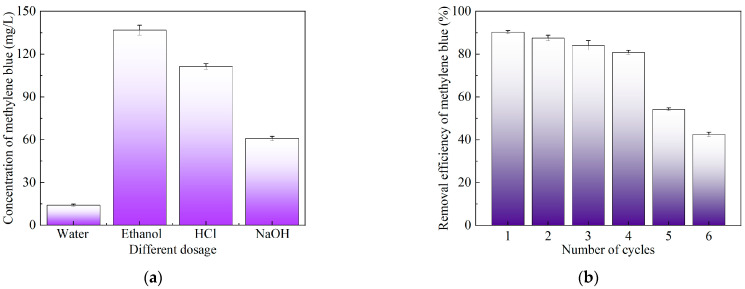
Reusability of Mg@FA. (**a**) Desorption effectiveness of different eluents. (**b**) Effect of different adsorption–desorption cycle numbers on adsorption of methylene blue by Mg@FA.

From Figure 5a, it can be seen that the desorption effectiveness of different eluents (deionized water, anhydrous ethanol, 0.1 mol/L hydrochloric acid solution, and 0.1 mol/L sodium hydroxide solution) for methylene blue adsorbed on Mg@FA is as follows: anhydrous ethanol > 0.1 mol/L HCl > 0.1 mol/L NaOH > deionized water. This indicates that anhydrous ethanol is more suitable as an adsorbent for methylene blue on Mg@FA. From Figure 5b, it can be seen that as the number of adsorption–desorption cycles increases, the adsorption capacity of Mg@FA for methylene blue gradually decreases. After one adsorption–desorption cycle, the removal efficiency of methylene blue by Mg@FA is 90.26%. However, after five adsorption–desorption cycles, the removal efficiency of methylene blue by Mg@FA drops to only 54.18%. Despite the decrease in adsorption capacity after regeneration, Mg@FA still shows adsorption activity for methylene blue, successfully proving the reusability of Mg@FA. Even after the fourth adsorption–desorption cycle, the removal efficiency of methylene blue by Mg@FA is still >80%, demonstrating the potential for the sustained application of the Mg@FA adsorbent.

## 3. Materials and Methods

### 3.1. Experimental Materials

Fly ash was taken from a power plant in Fuxin, Liaoning Province. It is the solid fine ash produced after coal combustion in power plants. The fly ash contains SiO_2_, Al_2_O_3_, CaO, Fe_2_O_3_, K_2_O, Na_2_O, and other components, which account for 67.1%, 19.7%, 4.0%, 3.4%, 1.3%, 1.1%, and 3.4%, respectively [19]. This means that fly ash contains a large amount of inorganic cations (such as Na^+^, K^+^, Al^3+^, etc.). Magnesium ions have strong exchange capacity, and when modifying fly ash with magnesium, magnesium ions can exchange with these cations [17,18]. This facilitates the incorporation of more magnesium ions into the internal layers of the fly ash, causing the lamellar structure to gradually peel apart, exposing more negative charges [34], thus promoting the adsorption of methylene blue dye. Methylene blue, MgCl_2_, NaOH, and other term chemicals were purchased from Tianjin dengfeng chemical reagent factory (Tianjin, China). Moreover, 10 g, 80–100 mesh fly ash was added to 100 mL, 1.5 mol/L MgCl_2_ solution. It was stirred for 30 min in a constant temperature magnetic stirrer (DS/ZNCL type, Zhengzhou Dongsheng Instrument and Equipment Co., Ltd., Zhengzhou, China) at 25 °C and 150 r/min. Then, 100 mL of 2.5 mg/L NaOH solution was added. It was continuously stirred with a constant temperature magnetic stirrer for 12 h and then stood at 25 °C for 24 h. The sediment was washed 5 times with deionized water and dried at 105 °C for 48 h in a blast drying oven (GZX-9246MBE, Shanghai Boxun Medical Biological Instrument Co., Ltd., Shanghai, China). The dried samples were baked in a muffle furnace (SX2-5-12, Shanghai Leiyun Test Instrument Manufacturing Co., Ltd., Shanghai, China) at 450 °C for 2 h to obtain Mg@FA.

### 3.2. Experimental Methods

Through a series of adsorption experiments (Table 5), the effects of Mg@FA dosage, adsorption time, and methylene blue concentration on the adsorption of methylene blue by Mg@FA were analyzed. Specifically, Mg@FA was added to the methylene blue solution according to the parameters in Table 5, and adsorption was carried out at 25 °C and 150 rpm for a certain period of time. After adsorption, the solution was filtered using a 0.22 μm filter membrane, and the remaining concentration of methylene blue was measured using a spectrophotometer (V-1600PC model, Shanghai Jingke Industrial Co., Ltd., Shanghai, China) at a wavelength of 665 nm. Each experiment was repeated three times, and the average value was used to plot the graph. The adsorption capacity and removal efficiency of Mg@FA for methylene blue were calculated. In the single-factor experiment, 1 g of Mg@FA was added to 100 mL of 150 mg/L methylene blue solution, and adsorption was carried out at 25 °C and 150 rpm for 120 min. The remaining concentration of methylene blue was measured to analyze the effect of pH on the adsorption of methylene blue by Mg@FA. Meanwhile, the zero charge point of Mg@FA was determined using the pH drift method. The background electrolyte was 0.1 mol/L NaCl, and the initial pH (pH_i_) of the solution was adjusted to 3–10 using 0.1 mol/L NaOH or HCl. Mg@FA was added to NaCl solutions with different initial pH values, and the pH value at equilibrium (pH_f_) was measured. A plot of the relationship between ΔpH(pH_f_–pH_i_) and pH_i_ was then constructed. Based on the results of batch adsorption experiments, the Lagergren first-order kinetic equation (Equation (1)), Lagergren second-order kinetic equation (Equation (2)), the intraparticle diffusion model (Equation (3)), Langmuir adsorption isotherm model (Equation (4)), and Freundlich adsorption isotherm model (Equation (5)) [35] were used to fit the results, and the adsorption kinetics and adsorption isotherms of Mg@FA on methylene blue were analyzed.

(1) $$q_{t}=q_{e}\times \left(1-exp\left(-k_{1}\times t\right)\right)$$

(2) $$q_{t}=\frac{k_{2}\times {q_{e}}^{2}\times t}{1+k_{2}\times q_{e}\times t}$$

(3) $$q_{t}=k_{p}\times t^{0.5}+c$$

(4) $${\frac{C_{e}}{q_{e}}=\frac{1}{q_{m}K}}_{L}+\frac{C_{e}}{q_{m}}$$

(5) $${lnq}_{e}={lnK}_{F}+\frac{1}{n}{lnC}_{e}$$

where *q*_t_ (mg/g) is the adsorption capacity of Mg@FA for methylene blue at *t* (min). *q*_e_ (mg/g) is the adsorption capacity of Mg@FA for methylene blue. *k*_1_ (min^−1^) and *k*_2_ (mg/(g·min)) are the adsorption rate constants of Lagergren first-order and second-order kinetic models, respectively. *kp* (mg/(g·min^0.5^)) is the intraparticle diffusion rate constant for Mg@FA adsorption of methylene blue; and *c* (mg/g) is the parameter related to the boundary layer. *C*_e_ (mg/L) is the equilibrium concentration of methylene blue. *q*_m_ (mg/g) is the adsorption capacity of Mg@FA for methylene blue at the adsorption equilibrium state. *K_L_* (L/mg) and *K_F_* (mg^(1−1/n)^·L^1/n^·g^−1^) are the adsorption constants of the Langmuir and Freundlich models, respectively. *n* is the adsorption strength constant of Mg@FA for methylene blue in Freundlich model.

To regenerate and recycle Mg@FA, 1 g of Mg@FA was added to 150 mg/L methylene blue solution at a ratio of 10 g:1 L, and adsorption was carried out at 25 °C and 150 r/min for 180 min until adsorption saturation was reached. The Mg@FA adsorbed with methylene blue was then added to 0.1 L of the desorption agents (deionized water, anhydrous ethanol, hydrochloric acid solution, sodium hydroxide solution) at the same ratio of 10 g:1 L, and soaked for 6 h for adsorbent regeneration [36]. The desorption agent was filtered using a 0.22 μm filter membrane, and the methylene blue concentration in the desorption agent was measured at a wavelength of 665 nm. By comparing the methylene blue concentrations in the desorption agents, the desorption effects of different agents were analyzed. The desorption agent with the best effect was then selected for the regeneration ability study of Mg@FA. After regeneration, the Mg@FA was separated, washed five times with deionized water, and reused. Six adsorption–desorption cycles were performed to analyze the regeneration and recycling performance of Mg@FA.

## 4. Conclusions

In this study, a new adsorbent Mg@FA was prepared by using fly ash as raw material to remove methylene blue. Mg@FA has the following characteristics: when the dosage of Mg@FA is 1.0 g, the adsorption time is 120 min, and the initial concentration of methylene blue is 150 mg/L, the removal efficiency and adsorption capacity of Mg@FA for methylene blue are 95.61% and 14.34 mg/g, respectively. Compared to untreated fly ash, the Mg@FA prepared in this study showed an 83.08% increase in removal efficiency of methylene blue, with an adsorption capacity 7.63 times that of fly ash. When the pH of the methylene blue solution was between 5 and 11, Mg@FA, which is negatively charged, formed electrostatic attractions with methylene blue, facilitating its adsorption. The adsorption of methylene blue by Mg@FA follows the second-order kinetic model and the Langmuir model. The adsorption process of methylene blue by Mg@FA is spontaneous and endothermic. Mg@FA adsorbs methylene blue through electrostatic interaction and hydrogen bonding. Mg@FA provides a new method for the reuse of fly ash and the fixation of methylene blue dye, which can realize the treatment of waste by waste, and is a sustainable dye wastewater treatment material.

## Figures and Tables

**Table 1:** Adsorption of methylene blue by different adsorbents.

Adsorbent	Adsorbent Dosage (g:mL)	Methylene Blue Concentration (mg/L)	Adsorption Capacity (mg/g)	Adsorption Removal Efficiency (%)	Refs.
Mg@FA	1:100	150	14.34	95.61	this study
Fly ash	2:100	0.5	0.12		[5]
Fly ash	0.6:30	100	5.718	-	[21]
Synthesized calcium silicate nanopowders from waste materials	0.5:1000	10	13.44	-	[22]
Starch phosphate grafted polyvinyl imidazole	0.05:20	100	5.2	95.4	[23]
Magnetic/activated charcoal/β cyclodextrin/alginate polymer beads	0.2:10	5	2.079	99.53	[24]
Walnut shell-activated carbon	0.1:50	-	3.53	-	[25]
Almond shell-activated carbon	0.1:50	-	1.33	-	[25]
Corn cob-derived activated carbon	0.3:0.6	-	0.84	-	[26]
Fir wood-derived activated carbon	0.1:0.1	200	1.21	-	[27]
Magnetized corn cobs	4:1000	25	13.23	-	[28]
Biochar prepared from date palm seeds	2:100	10	40.76	85.6	[29]

**Table 2:** Kinetic fitting data of adsorption of methylene blue by Mg@FA.

Adsorption Kinetics Model	Parameter	Adsorption of Methylene Blue by Mg@FA
Pseudo-first-order kinetic model	*q*_e_ (mg/g)	14.33689
	*k*_1_ (min^−1^)	0.03715
	*R* ^2^	0.95354
Pseudo-second-order kinetic model	*q*_e_ (mg/g)	15.32332
	*k*_2_ (mg/(g·min))	0.00444
	*R* ^2^	0.99859
Intraparticle diffusion model	*k*_p1_	0.91424
	R^2^	0.95843
	*k*_p2_	0.01668
	R^2^	0.95230

**Table 3:** Freundlich and Langmuir fitting data of Mg@FA adsorbing methylene blue.

*T* (K)	Langmuir Model				Freundlich Model		
	*q*_m_ (mg/g)	*K_L_* (L/mg)	*R* ^2^	*R*_L_	*K_F_* (mg^(1−1/n)^·L^1/n^·g^−1^)	*n*	*R* ^2^
298	13.77221	1.34115	0.99801	0.00297–0.00984	6.67894	5.36653	0.43402
303	14.26941	2.01321	0.99802	0.00198–0.00919	6.95840	5.23780	0.51535
308	14.53488	2.15742	0.99860	0.00185–0.00919	7.15056	5.24934	0.56518

**Table 4:** Thermodynamic parameters of Mg@FA adsorption for methylene blue.

*T* (K)	∆G (kJ/mol)	∆H (kJ/mol)	∆S (kJ/(mol·K))
298	−0.72724	36.41429	125.08455
303	−1.76272		
308	−1.96896		

**Table 5:** Batch test parameters of Mg@FA adsorption of methylene blue.

Numbering	Experimental Factors	Experimental Condition			
		Mg@FA Dosage (g)	The Initial Concentration of Methylene Blue (mg/L)	Methylene Blue Solution Volume (mL)	Adsorption Time (min)
1	Mg@FA dosage	0.5, 1.0,1.5, 2.0, 2.5	150	100	120
2	Adsorption time	1	150	100	20, 40, 60, 120, 180, 240, 300
3	The initial concentration of methylene blue	1	50, 100, 150, 200, 250	100	120

## Data Availability

All the data were included in the study.
